# A Marketing Perspective on Disseminating Evidence-based Approaches to Disease Prevention and Health Promotion

**Published:** 2006-06-15

**Authors:** Edward W Maibach, Mary Ann S. Van Duyn, Bonny Bloodgood

**Affiliations:** Public Health Communication & Marketing Program, School of Public Health and Health Services, The George Washington University; National Cancer Institute, Bethesda, Md; National Cancer Institute, Bethesda, Md

## Abstract

Evidence-based disease prevention practice guidelines can provide a rationale for health programming decisions, which should, in turn, lead to improved public health outcomes. This logic has stimulated the creation of a growing number of evidence-based prevention practice guidelines, including the *Guide to Community Preventive Services.* Few systematic efforts have been made to document the degree of adoption and implementation of these approaches, although the evidence on translation of research into practice in other health fields indicates that the adoption and implementation rate is low. Drawing on the marketing literature, we suggest three approaches to enhance the adoption and implementation of evidence-based approaches: 1) conducting consumer research with prospective adopters to identify their perspectives on how evidence-based prevention programs can advance their organization's mission, 2) building sustainable distribution channels to promote and deliver evidence-based programs to prospective adopters, and 3) improving access to easily implemented programs that are consistent with evidence-based guidelines. Newly emerging paradigms of prevention research (e.g., RE-AIM) that are more attuned to the needs of the marketplace will likely yield a new generation of evidence-based preventive approaches that can be more effectively disseminated. We suggest that the public health community prioritize the dissemination of evidence-based prevention approaches, because doing so is a potent environmental change strategy for enhancing health.

## Introduction

The long, slow march toward the practice of evidence-based medicine began nearly a century ago with the publication of the Flexner report (*Medical Education in the United States and Canada*) (1), and the pace of the march has escalated in recent years (2). Government health organizations, health care professional societies, health care quality improvement organizations, voluntary health associations, and many insurers and payers are actively engaged in developing evidence-based practice guidelines and promoting their adoption (3-5). A similar movement toward the practice of evidence-based public health has emerged in recent years, including the development of evidence-based disease prevention practice guidelines (6).

Evidence-based disease prevention practice guidelines — in other words, syntheses of the prevention literature based on formal criteria to assess the level of evidence (7) — are the logical culmination of the health community's investment in prevention research in that they can provide a rationale for public health program decision making at the local, state, and national levels. The *Guide to Community Preventive Services* (the *Community Guide*) (8) and the *Guide to Clinical Preventive Services* (9) are perhaps the two most prominent examples of guidelines in the United States today, but similar resources can be found in developed and less developed countries around the world (e.g., from the Third Joint Task Force of the European and Other Societies) (10,11). In addition to their role in shaping public health decisions, these guidelines play a valuable role in disease prevention programming decisions that are beyond the bounds of the public health system at large, including decisions made by employers, community organizations, and potentially even consumers (12).

Currently, little empirical evidence documents the degree of adoption and implementation of these evidence-based approaches to prevention programming, although the preponderance of evidence on translating research into practice in other health fields suggests that rates of adoption and implementation are low (13-15). Brownson et al (16) recently conducted a study among physical activity contacts in all U.S. state and territorial health departments to determine their awareness and use of the evidence-based physical activity guidelines in the *Community Guide.* Among this highly motivated audience, the investigators found high rates of awareness of the physical activity guidelines (90%), somewhat lower rates of having read the *Community Guide* online (67%) and printing materials from its Web site (48%), and markedly lower rates of having modified existing programs (22%) or developed new programs (36%) based on information in the guide.

Relatively little research has been done on how to effectively disseminate evidence-based practices (17). For example, a recent review conducted by the Agency for Healthcare Research and Quality found fewer than 30 articles in the published literature on dissemination of cancer control interventions (18). Most of these studies were uncontrolled or descriptive.

The term *dissemination* is considered by some in the public health community to be part of the problem. To many in the public health community, disseminating means alerting audiences — often through journal articles — to new information. This concept of dissemination is consistent with *Merriam-Webster's* first definition of dissemination: "to spread abroad as though sowing seed" (19). However, we use the term dissemination to mean a series of planned activities intended to encourage and enable adoption and implementation of proven approaches. The Social Sciences and Humanities Research Council of Canada has suggested the term *knowledge mobilization* as a distinct and therefore potentially more useful term for this concept (20). Regardless of the term used — dissemination or knowledge mobilization — the marketing literature offers important insights into steps that can be taken to improve the adoption and implementation of evidence-based approaches to disease prevention.

This article draws on the marketing literature to suggest three approaches that can be implemented to enhance the dissemination of evidence-based approaches to disease prevention programming.

## Marketing Concepts

Marketing is a population-based behavior management strategy (21). Public health program managers can use consumer marketing to "manage" the health behaviors of people by providing them with healthier behavior options that are more attractive than other options in the marketplace (22,23). There is considerable precedent for the use of consumer marketing in the public health field, which is typically referred to as *social marketing*.

A second, less-explored use of marketing in public health is called *business marketing* (or *business-to-business marketing*) (24-26) and is relevant to the challenge of disseminating evidence-based preventive approaches (27). The objective of business marketing is to manage the behavior of businesses, in other words, of organizations rather than individuals. The fundamental strategy is to encourage cooperation among the businesses that are needed to bring a product (e.g., an evidence-based prevention program) to the marketplace. The Figure illustrates a hypothetical business marketing perspective on opportunities to distribute evidence-based approaches to obesity control.

**Figure.**
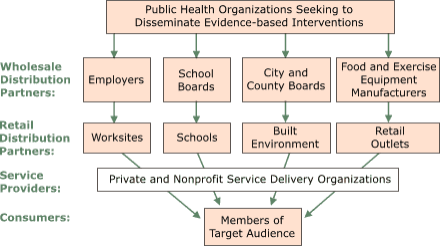
Potential distribution channels for evidence-based obesity interventions.

Marketing occurs when an organization makes an "offer" to its potential consumers: "Buy and use my product because it will improve your ability to run a profitable business." The offer is an attempt to induce prospective consumers, whether they are individuals, businesses, schools, nonprofit community-based organizations (CBOs), or government agencies, to purchase (or adopt if no purchase is required) and use (or implement) a product, service, or idea. When potential consumers consider the offer to be more attractive (i.e., to have more benefits, fewer costs, or both) than their other available options (i.e., the competing offers), they are likely to modify their behavior to purchase or adopt that option. Therefore, marketing organizations attempt to create an offer, whether it is a product, a service, or some other bundle of benefits, that can successfully compete in at least one segment of the marketplace.

Self-interest is a pivotal marketing concept. More than 200 years ago, Adam Smith noted that self-interest is the invisible guiding hand that ensures the efficiency of the marketplace (28). Smith's point was that individuals and organizations are usually willing to expend their resources — money, time, and effort — when they believe that doing so will advance their interests to an extent that is commensurate with the investment.

Marketers use consumer research to identify the ways in which prospective consumers define their self-interest (29). Insight gained from consumer research is then used to develop products (possibly requiring an investment in product research and development) and determine how best to price, promote, and distribute the products to maximize the odds of their success in the marketplace (22).

Marketing or distribution channels, also referred to as *place* in the *4 P's of marketing *(product, price, place, and promotion), are defined as "a set of interdependent organizations involved in the process of making a product or service available for use or consumption" (30) and are generally considered by commercial marketers to be the most important element in the marketing mix.

If one looks realistically at the major strategy variables of the marketing mix — product, price, promotion, and distribution — the greatest potential for achieving a competitive advantage (in the commercial sector) now lies in distribution (31).

David Heymann, the director of the World Health Organization's Polio Eradication Initiative, highlighted this point: "Coca-Cola, usually cold, can be found in nearly every village in every corner of the globe, no matter how remote" (32). Considerable attention has been paid to marketing channel development for certain disease-prevention products in less-developed countries (e.g., condoms, mosquito netting, hand soap) (33-37), yet the current need for developing marketing channels for evidence-based prevention programs in the United States is often overlooked.

Marketing approaches have much to offer the public health community in our efforts to disseminate evidence-based preventive approaches (38), and we describe some of these opportunities in the following sections. We differentiate between marketing the current generation of evidence-based approaches and using marketing to influence the development and dissemination of future generations of evidence-based approaches.

## Using Marketing to Improve Dissemination of Current Evidence-based Approaches

Three marketing approaches can be valuable in improving our efforts to disseminate current evidence-based approaches to disease prevention: 1) consumer research, 2) systematic efforts to build sustainable distribution channels, and 3) improved product and service development or selection.

### Consumer research

Current evidence-based disease prevention guidelines were developed largely without attention to the perceived self-interests of their prospective consumers: organizations that are potential adopters (i.e., potential distribution partners) and individuals who are potential users (i.e., potential program participants). To more effectively market these guidelines and improve their dissemination, we must better understand their benefits and costs through the eyes of potential consumers. With information about consumers' perceived benefits and costs, public health organizations interested in disseminating the guidelines can do so in a way that emphasizes the benefits most valued by consumers and recommends ways of reducing or altogether avoiding the costs that are of greatest concern to them.

For example, consumer research is being conducted by several grantees of the National Cancer Institute's (NCI's) Special Populations Network to determine how best to promote evidence-based physical activity guidelines in communities with health disparities (39). Specifically, they are conducting market research with members of various communities (i.e., low-income Hispanic women, Hmong parents and their children, low-income African American women, and young adult Native Hawaiians) to identify consumers' perceived benefits of and barriers to participating in prevention programs and to identify relevant perceptions among organizations and individuals in a position to influence the adoption of evidence-based prevention programs in these communities (e.g., community health advisors, Native Hawaiian elders). Findings from this research will be used to develop and deliver evidence-based physical activity programming in ways that are attractive to and embraced by members of these communities.

Recent work by Bachman et al (40) provides a second example of consumer research conducted with potential wholesale (i.e., employers) and retail (i.e., employees) consumers of evidence-based workplace fruit and vegetable and physical activity promotion programs. They conducted telephone interviews with 40 managers of small, medium, and large California businesses and 12 focus groups among employed low- and middle-income women in California from diverse racial and ethnic backgrounds. They found that employees and managers were supportive of measures to promote enhanced nutrition and physical activity and that lack of easy access in the workplace was considered a major obstacle to both behaviors. Based on these findings, the California Department of Health is focusing its efforts on 1) improving access to healthy foods and physical activity at workplaces, such as offering healthier foods in vending machines, catering trucks, and cafeterias and including fruits and vegetables at meetings and other gatherings; and 2) fostering supportive work environments that encourage healthy lifestyles, such as organizing walking clubs for break times and providing access to showers and changing facilities. Consumer research allowed these public health managers to select and customize the evidence-based approaches that were most applicable to their settings.

### Building sustainable distribution channels

Ultimately, the most important opportunity for moving evidence-based preventive approaches into the marketplace is the creation of sustainable distribution channels. The success and sustainability of these distribution channels requires all distribution partners to advance their mission (or some other aspect of perceived self-interest) by performing the role required of them in the distribution process.

For example, consider local YMCAs and their national organization as potential distribution partners for evidence-based physical activity guidelines and related programming. Through nearly 2500 retail outlets, the YMCA is one of the largest vendors of physical activity programming in communities across the United States. Public health organizations seeking to promote evidence-based physical activity programming should consider the YMCA's managers, program planners, and other physical activity staff members as potential distribution partners for evidence-based physical activity programming. Indeed, a similar partnership began recently through the YMCA's Activate America initiative. The initiative involves working with partners from multiple sectors of society to create a culture and national movement to support healthy lifestyles, all with a foundation in evidence-based approaches (41). The initiative is an opportunity for the YMCA and national public health agencies to create a powerful and sustainable distribution channel for evidence-based preventive approaches.

Kelder et al's efforts to disseminate the Child and Adolescent Trial for Cardiovascular Health (CATCH) — an evidence-based school physical activity promotion and nutrition intervention — to elementary schools in Texas is another example of how to build a distribution channel for evidence-based preventive approaches (42,43). Several years after publication of the article that demonstrated CATCH's effectiveness in Texas and three other states (44), only a few schools in Texas had adopted the program (i.e., the schools that had participated in the efficacy trial). A dissemination team was formed, which developed a training module to teach elementary school officials how to implement CATCH. The team also determined how best to promote CATCH among decision makers. Harnessing the precepts of diffusion theory (45), the team decided to emphasize that CATCH 1) has *advantages* over other approaches available to elementary schools, 2) is *compatible* with the ongoing operations of Texas elementary schools, 3) is *not complicated* to implement because it modifies rather than replaces what schools are already doing, 4) is able to be implemented on a *trial* basis because the start-up costs are minimal, and 5) will create *observable* beneficial changes in the school environment and in student health. Also following the precepts of diffusion theory, the dissemination team identified school health and physical activity opinion leaders within the Texas elementary school system as initial recruits to receive the training. These initial adopters — by virtue of their word-of-mouth recommendations to peers and their nominations of peers for training — created considerable demand among officials in other elementary schools for the University of Texas CATCH training.

Despite minimal levels of funding, Kelder et al's approach to building a CATCH distribution channel has been highly successful. CATCH has been implemented in more than 1500 schools, or approximately 30% of all Texas elementary schools (46). Although retention rates by schools have been high because the program is relatively easy to sustain and has been well-received by principals, teachers, and students, the dissemination team identified staff training, having a program champion, and having adequate administrative support and resources as being critical to continued participation (47).

Glanz et al's Pool Cool program, a skin cancer prevention program delivered in aquatic settings, is yet another example of distribution channel development (42). Pool Cool began as a marketing- and theory-based skin cancer prevention intervention, with pilot studies involving aquatics staff members and pool managers in Hawaii and Massachusetts (48). Informed by the pilot study finding, Glanz et al conducted a randomized trial at 28 pools in Hawaii and Massachusetts that resulted in significant changes in multiple sun-protection behaviors, including use of sunscreen and shade among children as well as improvements in parents' sun-protection habits and reported sun-protection policies and pool environments. Encouraged by these findings, the researchers successfully developed a partnership with the National Recreation and Parks Association — whose members operate a large proportion of U.S. public pools —  to conduct an initial dissemination pilot of Pool Cool among 186 pools and a second dissemination pilot among 282 pools. Today, with funding from NCI, the dissemination of the Pool Cool program continues to be enhanced through a diffusion trial in more than 400 pools nationwide. This well-integrated approach to intervention development and validation and subsequent distribution provides a sound model for developing distribution channels to translate research into practice.

### Improving products and product selection and reducing product price

By necessity, evidence-based guidelines are broadly stated descriptions of approaches that have been shown to be effective in numerous independent tests; they are not specific programs, products, or services. However, wholesale consumers for evidence-based approaches (e.g., employers, community-based organizations) cannot offer guidelines per se to their consumers (e.g., employees, members); they must offer actual products or services. The type of consumer research described in the previous section can be used to identify how best to create or select from among existing products and services those that are consistent with evidence-based guidelines *and* provide wholesale and retail consumers with an attractive bundle of benefits at an acceptable cost.

The California Department of Health Services (CDHS) used insight gained from consumer research to reduce the price of fruits and vegetables at farmers' markets for residents with low incomes (49). Previously, vendors at farmers' markets — of which there are many throughout California — did not accept food stamps because the farmers' markets were not set up to handle electronic balance-transfer cards (which are the way food stamp funds are distributed in California). CDHS's consumer research with food stamp recipients identified the inability to use food stamps at farmers' markets as an important barrier to purchasing items. CDHS responded by using their resources to deploy electronic balance transfer (EBT) card readers to select farmers' markets. This EBT card program is an innovative and successful effort to increase access to fruits and vegetables among food stamp recipients by removing a purchase barrier. Food stamp recipients appreciate their enhanced access to fresh produce, and vendors like the technology because it allows them to apply EBT receipts toward their stall fees. Based on the success of this innovation, farmers' markets in California are now exploring the adoption of this technology for all types of electronic banking, including debit and credit cards, to enhance efficiency of their market.

NCI, in partnership with the Centers for Disease Control and Prevention (CDC), American Cancer Society, Substance Abuse and Mental Health Administration, and Agency for Healthcare Research and Quality, created an online marketplace to enhance the use of research data and evidence and facilitate the selection of evidence-based cancer prevention programs that are available for adoption by wholesale distributors (e.g., state and county health departments, employers). Cancer Control PLANET (www.cancercontrolplanet.gov) is a Web site that offers users the ability to review cancer incidence, mortality, and risk factor data; identify research and program partners; explore systematic reviews of research evidence (e.g., the *Community Guide*); and find evidence-based cancer control programs and products that are available for use by other organizations (50). NCI and its partners are actively encouraging their grantees to populate PLANET's library of products and services with prevention programs that have been proven to be effective. As the number of programs available for adoption continues to grow, PLANET can play an important role in encouraging organizations to adopt proven disease-prevention programs rather than create new or adopt unproven programs. Although the evidence-based products and services listed in PLANET are not currently categorized based on the categories of evidence-based approaches identified in the *Community Guide,* this modification is being designed for future versions of PLANET.

## Using Marketing for the Next Generation of Evidence-based Approaches

The public health field needs to begin funding disease prevention research that will yield evidence-based guidelines and specific products and services that members of the marketplace — distributors and consumers — will be eager to embrace. In other words, rather than developing proven approaches and then retrofitting them to the dynamics of distributor and consumer preferences, a better approach would be to develop and test programs that specifically respond to consumer and distributor preferences. The synthesis of this research over time will yield the next generation of evidence-based approaches to prevention, approaches that can be more effectively disseminated because they were created by assessing needs in the marketplace.

The RE-AIM model created by Glasgow et al provides a systematic framework for evaluating the potential public health impact of current prevention research (51-54). RE-AIM can also be thought of as a framework for evaluating the dissemination potential of prevention research. RE-AIM examines individual (i.e., end-user) and institutional (i.e., distributor) impact factors associated with the success or failure of preventive interventions. Specifically, the end-user impact factors are *reach* (i.e., the proportion of people who accepted the "offer" made to them) and *effectiveness* (i.e., the impact of the offer on their behavior). The distributor impact factors are *adoption* (i.e., the percentage of potential distribution partners that choose to participate), *implementation* (i.e., the effectiveness of distribution partners in delivering all components of the offer), and *maintenance* (i.e., the proportion of distribution partners who continue as long-term distributors of the offer).

From a marketing perspective, the RE-AIM framework can be used to guide research and development efforts to create disease-prevention interventions. Before development of the preventive intervention, consumer research can be conducted with targeted consumers to gain insight about how to create the offer in a way that will maximize reach. Similarly, consumer research can be conducted with potential distributors to determine how to develop the offer in ways that will increase chances of adoption and improve implementation and maintenance.

In a critical appraisal of the current paradigm for preventive intervention research, Rotheram-Borus et al (55,56) conclude that the next generation of preventive interventions should be driven by private enterprise models, or marketing-based approaches, because of their enhanced potential for widespread adoption and maintenance. Their suggestions to facilitate the development of these next-generation preventive interventions include modifying the composition of research teams (and the training of principal investigators) to include a marketing and business development focus; using marketing research to improve the acceptability of program design features to consumers, providers, and funding agencies; developing national marketing surveys to identify viable target markets and opportunities; building programs to be robust enough for implementation by many kinds of people and organizations and flexible enough to evolve over time as evidence accumulates to support program modification; branding these programs to heighten consumer demand; and having programs certified by an independent credible organization to increase consumer and provider trust.

Although arising from an entirely different public health perspective, community-based participatory research (CBPR) has many attributes of a marketing-based approach to disease-prevention research. CBPR incorporates collaborative partnership approaches to actively involve participants in all phases of the research process from problem identification through adoption and dissemination of results (57). CBPR inherently recognizes the community as an integral partner in the research endeavor; the knowledge and experiences of community members are incorporated into the research process to ensure acceptance and promote improved community health (58). For example, CDHS used a CBPR approach in the planning and implementation of the California Health Interview Survey (59). By including community members from advisory boards, technical advisory committees, and work groups, the quality of the survey improved. Moreover, collaborative processes help ensure that the findings are considered relevant by and therefore useful for participating communities and their advocates.

## Disseminating Evidence-based Preventive Approaches

Even if all public health organizations completely adopted evidence-based approaches to community preventive services, we believe that they would have limits in their ability to accomplish the public health goals that have been established for our society (e.g., *Healthy People 2010* goals) (60). The organizations have neither the resources nor the opportunities to influence the lives of most people in a manner commensurate with our national health goals.

Ecological models of health emphasize that behavior and health are influenced by individual attributes as well as the conditions under which people live (61). People's environments influence their behavior and their health, independent of behavior. By altering people's environments in putative ways, we can create conditions conducive to good health (27). One important aspect of creating healthy environmental conditions is the creation of conditions under which it is as easy, if not easier, to behave in healthy rather than unhealthy ways. Unfortunately, many facets of our environment fall short of this ideal; it is often easier to be unhealthy, particularly for communities with the fewest economic resources and the most people with poor health.

Ecological models of health suggest that to achieve U.S. public health goals, we must cultivate and mobilize the contributions of the other sectors of society that influence our population's health. These sectors, especially the for-profit businesses and nonprofit community organizations that have the most frequent interaction with people and therefore the greatest potential to influence their lives, must specifically be cultivated as adopters of evidence-based public health practices. They are a vast resource through which to promote the public's health. The public health community has forged many effective partnerships with businesses and community organizations (62-65), but these efforts have typically focused on specific initiatives rather than on identifying broad opportunities for enhancing the adoption and successful implementation of proven approaches to prevention. For-profit and nonprofit organizations will be motivated to embrace evidence-based approaches to prevention, but only to the extent that they perceive doing so will help them improve the bottom line of their organization. The public health community must determine how to effectively make this case.

## Conclusion

Marketing is considered by some in the public health community to be a major part of public health's problem rather than part of the solution (66,67). The concerns about marketing are entirely understandable, because commercial marketing has played a major role in the creation of unhealthy environments (e.g., environments that encourage the consumption of tobacco, alcohol, and excess calories as well as sedentary behaviors) (68,69). The negative influence of marketing on public health is well documented for certain behaviors (e.g., tobacco, alcohol) (70,71) and less well documented but widely held for other behaviors and conditions (e.g., excess calorie consumption, sedentary behaviors, obesity). In short, marketing has negatively influenced community environments.

However, we interpret the literature on marketing's harmful contributions to public health as prima facie evidence of marketing's potential to manage behavior and shape community environments, for better or worse. Marketing can be used to enhance or detract from the public's well-being. We believe that the public health community has an obligation as well as a major opportunity to harness the value of marketing in aggressively disseminating evidence-based approaches to prevention, the fruits of the United State's investment in prevention research. As highlighted by the examples in this article and by many others, this is slowly beginning to happen.

Marketing can be used to improve the environment and social ecology of our communities in evidence-based ways. The use of marketing to enhance dissemination of evidence-based disease prevention guidelines and specific programs, products, and services that are consistent with these guidelines has considerable potential to change the social ecology of our communities and promote healthy living, directly and indirectly. We believe that significant public health resources should be dedicated to this strategy, including expanding the training opportunities available to the public health workforce beyond those already offered by the Turning Point initiative and others (7,32,43,61-64,70-74). We encourage our public health colleagues to consider what they can do to cultivate this opportunity.
